# What could a strengthened right to health bring to the post-2015 health development agenda?: interrogating the role of the minimum core concept in advancing essential global health needs

**DOI:** 10.1186/1472-698X-13-48

**Published:** 2013-12-01

**Authors:** Lisa Forman, Gorik Ooms, Audrey Chapman, Eric Friedman, Attiya Waris, Everaldo Lamprea, Moses Mulumba

**Affiliations:** 1 Dalla Lana School of Public Health, University of Toronto, 155 College Street, Toronto, Ontario M5T 3M7, Canada; 2 Institute of Tropical Medicine, Nationalestraat 155, 2000, Antwerp, Belgium; 3 University of Connecticut School of Medicine, 263 Farmington Avenue, Farmington, Connecticut 06030, USA; 4 Georgetown University Law Center, 600 New Jersey Avenue NW, Washington DC 20001, USA; 5 University of Nairobi, P. O. Box 30197 – 00100 Parklands Campus, Nairobi, Kenya; 6 Los Andes University, Cra 1 Nº 18A- 12, Bogota, Colombia; 7 Centre for Health, Human Rights and Development, Plot 614 Tufnell Drive, Kamwokya, PO Box 16617, Wandegeya, Kampala, Uganda

**Keywords:** Human rights, Right to health, Minimum core obligations, Millennium development goals, Post-2015 health development agenda, Global health, Equity

## Abstract

**Background:**

Global health institutions increasingly recognize that the right to health should guide the formulation of replacement goals for the Millennium Development Goals, which expire in 2015. However, the right to health’s contribution is undercut by the principle of progressive realization, which links provision of health services to available resources, permitting states to deny even basic levels of health coverage domestically and allowing international assistance for health to remain entirely discretionary.

**Discussion:**

To prevent progressive realization from undermining both domestic and international responsibilities towards health, international human rights law institutions developed the idea of non-derogable “minimum core” obligations to provide essential health services. While minimum core obligations have enjoyed some uptake in human rights practice and scholarship, their definition in international law fails to specify which health services should fall within their scope, or to specify wealthy country obligations to assist poorer countries. These definitional gaps undercut the capacity of minimum core obligations to protect essential health needs against inaction, austerity and illegitimate trade-offs in both domestic and global action. If the right to health is to effectively advance essential global health needs in these contexts, weaknesses within the minimum core concept must be resolved through innovative research on social, political and legal conceptualizations of essential health needs.

**Summary:**

We believe that if the minimum core concept is strengthened in these ways, it will produce a more feasible and grounded conception of legally prioritized health needs that could assist in advancing health equity, including by providing a framework rooted in legal obligations to guide the formulation of new health development goals, providing a baseline of essential health services to be protected as a matter of right against governmental claims of scarcity and inadequate international assistance, and empowering civil society to claim fulfillment of their essential health needs from domestic and global decision-makers.

## Background

Global health institutions increasingly recognize that the international human right to the highest attainable standard of physical and mental health (the right to health) provides key legal and ethical principles to guide the formulation of goals to replace the health-related Millennium Development Goals (MDGs) which expire in 2015 [1,2]. Yet the contribution of this right to global health policy and equity is limited by the principle of progressive realization, which on its face, conditions the provision of even essential and basic health needs on the availability of adequate resources. This limitation creates “a loophole large enough in practical terms to nullify the [*International Covenant on Economic, Social and Cultural Right’s*] guarantees” – namely, the possibility that governments will illegitimately claim lack of resources as the reason they have not met their obligations [3]. Even policy-makers acting in good faith are given little guidance regarding the extent to which the right to health can be delayed or denied in service of other pressing social needs or legitimate resource constraints. Nor does the *International Covenant on Economic, Social and Cultural Rights* [the Social Rights Covenant or ICESCR] provide clarity about the specific requirements of international duties of assistance and cooperation for wealthier countries to realize the right to health in other countries, a key deficiency given the dependence of many lower income countries on international funding to realize universal health coverage [4]. In this light, progressive realization may serve to permit almost any kind of health deprivations in the short term, an idea ostensibly at odds with the basic human rights imperative of protecting human life and dignity.

International human rights law institutions have attempted to compensate for the problems created by progressive realization within available resources in part by developing the norm that states hold irreducible “minimum core” obligations to provide basic and essential health services that are not subject to progressive realization. In particular, the United Nations Committee on Economic, Social and Cultural Rights [the Social Rights Committee] (a group of independent experts tasked with monitoring state implementation of the Social Rights Covenant) has emphasized that while progressive realization requires states to “move as expeditiously and effectively as possible towards” the full realization of rights [5], minimum core obligations place imperatives for action upon states not subject to the temporal and resource flexibilities permitted by progressive realization. The minimum core concept makes an important contribution to realizing the right to health by demarcating ‘basic’ and ‘essential’ health services as a baseline to be protected as a matter of right against governmental claims of scarcity, and by locating domestic and collective action to realize these needs within a legally binding framework. Yet, while minimum core obligations have enjoyed some uptake in human rights practice and scholarship [3,6-12], their definition in international law fails to specify the kinds of health services that should be considered to fall within the ambit of minimum core obligations, or to specify the international obligations that wealthier states hold to assist poorer countries to meet core obligations. These gaps significantly undercut the ambition of minimum core obligations to protect essential health needs against inaction, illegitimate trade-offs, austerity and resource scarcity. If the right to health is to meaningfully guide global health initiatives like the post-2015 health development agenda, then the conceptual framework it relies on must be clarified in these key domains.

In this article we explore the emerging debate on the post-2015 health development goals [13-16] and the role of the right to health in this regard, focusing on the potential contribution of minimum core obligations to advancing basic health needs within this framework. In part one, we explore the post-2015 development process and current considerations for health goals. In part two, we analyze state duties under the right to health relevant to post-2015 health goals, focusing on the strengths and weaknesses of the minimum core concept. In part three, we identify future research directions for strengthening the minimum core concept that respond to these key weaknesses. We close with thoughts about the potential role that a strengthened minimum core concept could play in advancing global health equity.

## Discussion

### The MDGs and the Post-2015 Health Agenda

In September 2000, 189 heads of state meeting at the United Nations headquarters in New York issued the Millennium Declaration as a repurposed vision of the organization for the 21^st^ century that committed member states to global action to advance peace, the environment, good governance and development [17]. In the last realm, states committed to “spare no effort to free our fellow men, women and children from the abject and dehumanizing conditions of extreme poverty,” and identified development targets to be reached by 2015 including in relation to poverty, hunger, access to water, education, child and maternal mortality, infectious disease and access to medicines [17]. The risk was considerable that these commitments would meet the fate of multiple other global commitments “easily made but seldom met” [18]. With the goal of avoiding these commitments fading into similar obscurity and a broader ambition of broadening the development discourse beyond a narrow focus on economic growth, a handful of high-ranking United Nations (UN) staff established and co-chaired a group of institutional experts to extract key targets from the Declaration and translate them into a free-standing category [19]. The outcome was eight major human development goals with eighteen targets and forty-eight indicators to be met by 2015. Three goals related directly to health – child mortality, maternal mortality and infectious disease. Other goals relating to health determinants like water and sanitation adopted health related targets, while increasing access to essential medicines was included as a target under the global partnership for development goal.

Considerable institutional energy has been devoted to promoting and monitoring the MDGs, including the creation of a MDG Gap Task Force to improve monitoring of MDG 8; the submission from 2005 of national MDG reports to the United Nations Development Program (UNDP); the creation of a UN Millennium Project to lay out a practical plan for achieving the MDG; and the launch in 2002 of a UN Millennium Campaign to support citizen participation in achieving the MDG. This system-wide effort has been dispersed through multiple international organizations, including five regional economic commissions and 29 international institutions including UN institutions such as the UNDP, WHO, Food and Agriculture Organization (FAO) and International Labour Organization (ILO), and other institutions such as the World Bank, International Monetary Fund and World Trade Organization [20]. As these examples suggest, these institutions have widely divergent agendas and missions, producing fractured initiatives, priorities and processes in advancing development goals. Moreover, the high rhetoric of the MDGs has not always been matched by actual institutional processes [21].

Concerns about the process and substance of the MDGs have seen them subjected to significant critiques: for being radically under-ambitious in seeking to only halve gross poverty and health deprivations [22]; for being created in a non-deliberative, non-transparent, non-inclusive top-down process [23,24]; for providing a “one-size fits all” approach through global benchmarks that are impervious to national conditions and disparities [25]; for being technocratic and reductionist, poorly specified, gender-blind and weakly accountable [26], and for permitting “cunning” low and middle income countries to ‘pass the buck’ for policy failures [27,28].

Given these critiques it is perhaps not surprising that the MDGs have produced inequitable outcomes. Goals on child mortality, water and infectious diseases have made significant progress, while those on maternal mortality, sanitation and essential medicines have seen limited and sometimes negligible gains [16,29-32]. Limited progress on maternal mortality, sanitation, and medicines underscores that collective health action is hamstrung not simply by the inadequacy of domestic and international resources, but by questionable trade-offs when remedial action is deemed to be of lesser importance to donors or to threaten prevailing social and economic power structures.

These uneven gains have motivated considerable efforts to capitalize on the strengths of the MDGs and remediate their substantive and procedural weaknesses. At the September 2010 MDG Summit, the United Nations launched a process designed to renew the MDGs after 2015, appointing a UN System Task Team to coordinate preparations and a “High-Level Panel of Eminent Persons” to advise the UN Secretary-General on the post-2015 development agenda. In addition, national consultations have been held in approximately 100 low and middle-income countries, alongside six regional consultations and 11 global thematic consultations (including on health). The High-Level Panel drew on these inputs to make recommendations on post-2015 goals for consideration at a UN General Assembly summit in September 2013 [15]. At the latter General Assembly, member states considered inputs from a range of sources including the High-Level Panel as well as the Sustainable Development Solutions Network, an advisory group launched in 2012 by the UN Secretary General to advise on the development of a set of Sustainable Development Goals (SDGs) that built on the MDGs as mandated at the “Rio + 20” UN Conference on Sustainable Development in 2012. Member states of the UN General Assembly decided to launch a process of intergovernmental negotiations over the next two years that will include inputs from civil society, academics, governments and the private sector, and culminate in a head of state and government summit in September 2015 when the post-2015 development agenda will be adopted [33]. This intergovernmental process is expected to converge with the “Rio + 20” mandated development of SDGs into a single universal development framework [34].

Earlier debates with regard to health had primarily focused on replacing the MDG’s disparate health goals and targets with one overarching health goal that would aim at achieving universal health coverage [13,14,16]. In 2013, discussion shifted to a goal of maximizing healthy life expectancy, which would subsume universal health coverage as a subsidiary target and also include a focus on underlying determinants of health [14,35]. The latter approach has been partially adopted by the High-Level Panel, which issued a report in May 2013 proposing a goal of ensuring healthy lives. However, instead of adopting a broad focus on universal health coverage through health care and the social determinants of health, the proposed goal focuses on five specific targets in relation to child mortality, maternal mortality, vaccinations, sexual and reproductive health rights and infectious and non-communicable disease [15]. It is unclear at this stage whether universal health coverage will be included in the final formulation of the goals, which will be decided through state negotiation at the UN over the next two years. It is notable however that universal health care (rather than coverage) is a recommended target under an overarching goal of “achieving health and wellbeing at all ages” made by the Sustainable Development Solutions Network [36], and was included as a primary component of health measures in the UN Secretary General’s July 2013 report which makes recommendations to the UN General Assembly on the post-2015 development agenda [37]. In addition, the concept continues to receive support from countries including the Oslo Seven (France, Norway, Indonesia, Senegal, South Africa, Brazil, and Thailand) [35], and institutions such as the WHO, which has been a primary mover of universal health coverage as an overarching post-2015 health goal [2,4].

Yet it is arguable that the failure to adopt the goal of universal health coverage is attributable at least in part to its nebulous definition. For example, the World Health Organization (WHO) defines universal health coverage as engaging the range of services available to people, the proportion of costs of services, and the proportion of the population covered [2]. Services are not defined beyond that they are “needed health services (prevention, promotion, treatment and rehabilitation)” [2]. Similar vagueness is apparent in a 2012 UN General Assembly resolution promoting universal health coverage, defining it as non-discriminatory access to “nationally determined sets of the needed promotive, preventive, curative and rehabilitative basic health services and essential, safe, affordable, effective and quality medicines” [16]. For universal health coverage or the broader goals of maximizing/ensuring healthy lives and achieving health and well-being at all ages to achieve their considerable ambitions, it is imperative that these concepts are adequately substantiated and that corresponding state responsibilities for domestic and international action are defined.

### The right to health and minimum core obligations

We suggest that the right to health in international law can assist in clarifying the content of the post-2015 health goals and in defining corresponding state responsibilities. International law has recognized individual rights and state obligations towards health at least since 1948, when the *Universal Declaration of Human Rights* recognized every person’s right to a standard of living adequate for their health and well-being, including medical care [38]*.* The most authoritative protection of the right to health is found in the the Social Rights Covenant, where state parties recognize everyone’s right to the enjoyment of the highest attainable standard of health, and agree to take a number of steps to achieve this including reducing infant mortality, addressing infectious disease and assuring medical service to all in sickness [39]. Subsequent treaties protect rights to health for specific populations, including racial minorities, women, children, migrant workers, and people with disabilities [40-43]. Rights to health are protected in each regional human rights system [44-46] and in at least 115 domestic constitutions globally [47,48].

This increase in legalization has contributed to a significant growth in the right to health’s legal, social and political force. At the domestic level, there has been a significant upsurge in right to health litigation over the last twenty years, especially in middle-income countries like Colombia, Brazil and Costa Rica [49,50]. A recently adopted “Optional Protocol” will give the Social Rights Committee the authority to review individual complaints of social rights violations, significantly augmenting the enforceability as well as interpretation of the right to health at the international level. The right to health has also played a central role in human rights activism including most prominently in relation to AIDS treatment [51] and sexual and reproductive health rights [52]. These developments have prompted growing recognition by scholars and policy-makers of the right to health’s contributions in relation to global economic forces and trade law, global health governance, social determinants of health and non-communicable disease [51-58]. Given the prominence of the MDG and post-MDG processes, there has also been considerable scholarship focused on initial divergences between human rights and the MDG process [24,59] and conversely, the contribution that human rights make to the MDGs and post-2015 process [60,61]. For example, scholars argue that human rights and the right to health could offer critical gains to the post-2015 agenda, including by providing accountability mechanisms; prescribing a participatory process for their development and ensuring a focus on non-discrimination and equality rather than on average improvements [60,61].

Yet the potential contribution of the right to health in these realms is hampered by the ICESCR’s limitation of state duties to progressively realizing this right within maximum available resources. ‘Progressive realization’ is the term of art used to describe a central element of the implementation article of the Social Rights Covenant which limits domestic state duties to realize social rights like health to taking steps, individually and through international assistance and cooperation, especially economic and technical, to the maximum of its available resources, with a view to achieving progressively the full realization of the rights recognized in the present Covenant by all appropriate means, including particularly the adoption of legislative measures [39].

In other words, domestic state duties under social rights like the right to health are limited to taking action over time within available resources, recognizing that social and economic rights like the right to health cannot generally be achieved quickly. A further weakness of the Covenant’s implementation article lies in its recognition that for many countries, social rights will be realized only in conjunction with international assistance. Yet this article is ambiguous as to whether rich countries hold a duty of international assistance, while more clearly placing a duty on poorer countries to seek such assistance. While international human rights law experts have since developed strong articulations of international responsibilities to assist in realizing social rights [62], progressive realization nonetheless continues to create a double-bind for the realization of the right to health. On the one hand, it undermines domestic responsibilities towards health by seemingly permitting countries to justify almost any level of health spending; on the other hand, it weakens the Covenant’s feebly articulated duties of international assistance.

#### The minimum core of the right to health

To clarify the extent to which progressive realization within resources could limit the right to health, in 2000 the Social Rights Committee issued a “general comment” that extensively interprets the right to health. The Committee issues general comments on various rights to guide states in fulfilling their corresponding duties. General comments are “soft law” instruments and not legally binding; however, they are very influential and it is expected that they will be respected [63]. In General Comment 14, the Committee endorsed its earlier suggestion that states hold “minimum core obligations” to ensure satisfaction of minimum essential levels for all [5]. The Committee had previously indicated that these minimum core obligations would include essential primary health care, essential foodstuffs, basic shelter and housing, and the most basic forms of education [5]. However essential primary health care was not defined, and in General Comment 14 for the first time, the Committee clarified the content of “core obligations” under the right to health. The Committee indicated that these core obligations include at least: (1) ensuring non-discriminatory access to health facilities, goods and services, especially for vulnerable or marginalized people, (2) ensuring access to food, basic shelter, housing, sanitation and water, (3) providing essential drugs as defined by WHO, (4) ensuring equitable distribution of all health facilities, goods and services and (5) adopting a national public health strategy and plan of action addressing the concerns of all [64]. The Committee also indicated that states hold “obligations of comparable priority” regarding reproductive, maternal and child health care, immunization against major infectious diseases, preventing, treating and controlling epidemic and endemic diseases, health education and access to information and appropriate training for health personnel [64].

The Committee’s effort to protect essential health needs against unreasonable restrictions in service of “progressive realization within resources” is apparent in its suggestion that minimum core obligations are non-derogable (i.e.: cannot be restricted or limited in any way) and that “a State party cannot, under any circumstances whatsoever, justify its non-compliance with the core obligations” [64]. This strong protection is similarly evident in the obligatory leverage that minimum core rights are deemed to create with regard to international assistance and cooperation. For example, the Committee emphasizes in General Comment 14, as in other comments on rights to water, work, and social security, that “it is particularly incumbent on States parties and other actors in a position to assist, to provide “international assistance and cooperation, especially economic and technical” which enable developing countries to fulfil their core and [comparable priority obligations]” [6-9,64].

#### Global uptake of minimum core obligations

There has been a relatively wide application of the concept of minimum core obligations by human rights institutions globally. The United Nations Committee on Economic, Social and Cultural Rights (Social Rights Committee) has used the concept of minimum core obligations in defining obligations under other social rights, including food, water, work, and social security [6-9]. The United Nations Committee on the Elimination of Discrimination against Women (which oversees the *International Covenant on the Elimination of All Forms of Discrimination against Women*) identifies non-discrimination as a core obligation under that Covenant [10] and found Brazil in violation of this duty for failing to assure appropriate maternal health services [11]. In addition, several domestic courts use the concept of minimum core obligations in adjudicating right to health claims against governments: The Colombian Constitutional Court (CCC) has distinguished an essential minimum core of the right to health based on the state’s mandatory health plan and ordered provision of a wide range of goods and services, including antiretrovirals and cancer medications [65-67]. Several rulings by the Costa Rican Supreme Court have defined the minimum core of the right to health to include life-saving health-care for people with HIV/AIDS [68-70]. While the Indian Supreme Court has not directly cited the minimum core concept, “the essential minimum” and “what is minimally required” have been primary considerations in claims for emergency medical care and minimum levels of food [71,72].

In the post-2015 health development context, human rights scholars and institutions are using the minimum core concept to argue for the provision of basic levels of health services and care as a global minimum standard. For example, the United Nations Office of the High Commission for Human Rights suggests that human rights treaties already obligate states to “aim for universal access to at least a basic level of social rights” [73]. The idea is similarly implicit in an international anti-poverty NGO’s suggestion that the post-2015 framework should be underpinned by “the idea of a social floor—a minimum standard below which people should not be expected to live” [74]. The implications of this floor for wealthier states is made explicit by the Go4Health consortium in which many of the authors of this piece participate, which argues that “there must be a floor of health care for all people, independent of the wealth of the countries they live in, for which the international community has responsibility” [75]. As a consortium and as individual scholars, they contend that minimum core obligations could assist in guiding universal health coverage by populating its definition of the services covered and the extent of population covered [75,76]. We suggest further that the prioritized and non-derogable nature of minimum core obligations could assist in expanding the proportion of costs covered under universal health coverage, since services demarcated as core would be prioritized in the allocation of resources.

#### The limitations of the committee’s definition of minimum core obligations

The potential contribution of the minimum core concept to advancing health equity is to demarcate “basic” and “essential” health services as a baseline to be protected as a matter of right against governmental claims of scarcity and against inadequate international assistance. Additionally, core obligations locate corresponding domestic and collective action within a legally binding framework with some enforceability and sometimes considerable normative and political influence. Yet the terse definition of minimum core obligations in General Comment 14 does not entirely support these lofty ambitions, and is marked by several key deficiencies.

First, beyond underscoring that health facilities, goods and services must be equally accessible and equitably distributed, the Comment provided little clarity on which health services, facilities and services fall within the minimum core beyond essential medicines and underlying determinants such as food, basic shelter, housing, sanitation and water [5,64]. While General Comment 14 reiterated an earlier general comment suggesting that states have a core obligation to ensure essential primary health care, it does not add definitional clarity to what constitutes essential primary health care beyond essential medicines, despite the fact that the Committee indicated that its conception of core obligations draws “compelling guidance” from contemporary instruments including the Programme of Action of the International Conference on Population and Development and the Declaration of Alma-Ata [64]. This lack of drafting and definitional clarity and the failure to specifically list primary care as one of the core obligations add confusion to whether in fact primary health care *is* considered to be a core obligation.

Second, while the Committee attempted to clarify the content of essential primary health by identifying “obligations of comparable priority,” the relationship between these and minimum core obligations remains unclear. If “obligations of comparable priority” are not minimum core obligations, do they reflect ‘next in line’ temporal priorities nonetheless subject to derogation through progressive realization and/or limited resources? The latter interpretation is supported by the Committee’s demarcation of these obligations not as core but as “comparable priority.” Yet it must be questioned whether a theory of the right to health that prioritizes essential medicines but not reproductive health care and infectious disease control is not flawed in terms of both human rights theory and public health practice [77].

Third, the Committee failed to explicitly identify international core obligations beyond telling richer states that it “is particularly incumbent” on them to provide international economic and technical assistance to developing countries towards meeting core and comparable priority obligations. Subsequent expert interpretations have attempted to resolve this gap by elaborating on the extraterritorial obligations that all states hold to respect, protect and fulfill economic, social and cultural rights [62]. In particular, states are obliged to prioritize the realization of “core obligations to realize minimum essential levels of economic, social and cultural rights” [12,62]. Yet international duties towards the minimum core remain significantly underspecified, with deleterious impacts on the realization of minimum core obligations in low and middle-income countries.

Fourth, the question of the resources necessary to meet minimum core obligations was not addressed in the Committee’s interpretations, beyond emphasizing that core obligations are non-derogable and that states cannot justify non-compliance with these obligations under any circumstances, including by implication, due to resource constraints [64]. While the injunction for richer states to provide international assistance and cooperation is presumably intended to fill in potential shortfalls towards realizing core obligations, the weakness of that imperative and the strong articulation of core obligations as non-derogable, appear to place financially unrealistic obligations on poorer countries.

Given these limitations and questions, the current conceptualization of minimum core obligations is widely viewed as incapable of supplying a predetermined content to the right to health and an inappropriate tool for judicial decision-making [78-80]. In addition, some human rights advocates worry about the risk that in practice, the minimum core concept will become a ceiling rather than a floor for health action that strips away non-core obligations under the right to health, notwithstanding the obligation to progressively fully realize the right to health [3]. Additional critiques come from social actors such as the People’s Health Movement which argues that even a well-defined essential health package comparable to core obligations will offer selective rather than comprehensive primary health care that threatens to reify stratified systems of health rights for the poor and rich [81,82]. Indeed, the Committee’s earlier delineation of “essential primary health care” as a core obligation begs the question of what constitutes “non-essential” primary care?

The conceptual and definitional limitations of the Committee’s definition of core obligations are apparent in South African case law, an important human rights jurisdiction with regard to the enforcement of social rights and the right to health in particular. Despite that country’s enforceable constitutional right to access health care services, South Africa’s Constitutional Court has rejected the minimum core concept because of resource constraints, the limited specificity of the Committee’s definition and a lack of legislative guidance [83-85]. It is arguable that the court’s rejection of minimum core obligations has had regressive outcomes for health-related needs: In a 2010 case, the Court rejected a claim by five Soweto residents that the City of Johannesburg’s free water allocations were insufficient and in violation of the state’s minimum core obligations [85]. Certainly, the South African judicial rejection of the core in favour of a “reasonableness standard” presents a significant challenge to the international law conception of minimum core obligations [51,86,87]. Significantly, however, while the South African Constitutional Court considers itself to lack the expertise necessary to define the core, it indicates that a better-defined core would assist in determining the reasonableness of governmental realization of social rights [83,84].

Conceptual vagueness similarly limits the core’s contribution to supporting international obligations, allowing rich countries to continue to provide inadequate support for minimum core obligations and to withdraw from existing commitments with impunity. Despite reaching an all-time high of USD28 billion per annum in 2010, international assistance for health remains insufficient to fund even existing health MDGs [88,89]. The inadequacy of current international health assistance suggests that ambitious health goals will not be achieved in these countries without a significant paradigm shift in the normative and legal underpinnings of international health assistance.

### Towards a feasible and grounded concept of minimum core obligations

We believe that if the core is to realize its potential of identifying essential health services within a binding legal framework, further research and scholarship is required to address the key weaknesses we have identified above. We argue that such work could include: (1) synthesizing existing judicial interpretations of the minimum core of the right to health and other social rights at various levels in order to establish how the concept is currently being interpreted and applied within legal systems globally; (2) identifying the content of packages of essential health benefits provided by the public health sector of governments at various income levels in order to establish current state practice with regard to the provision of essential health needs within available resources; (3) clarifying the conceptions and expectations of communities at various income levels with regard to essential health needs, in order to assure that minimum core conceptions derived from legal and policy sources are not abstracted from lived experience and needs; and (4) addressing the key question of the domestic and international resources necessary to realize core obligations. We believe that further research in these domains will enable the human rights community to build a more feasible and grounded conception of minimum core obligations in relation to essential health needs that can be applied within various contexts. In the remainder of the paper we explore two of these research directions in particular, namely public sector definitions of essential health benefits and the central question of resources.

We believe that research on essential health benefits packages will illustrate current policy approaches to “core” health needs, as well as illustrating pragmatically what low and middle income countries consider to be economically feasible in this regard. Many countries have defined national minimum packages of services using a variety of approaches to do so. A few have drawn from international proposals such as the primary health care approach outlined in the 1978 Alma-Ata Declaration [90] and (worryingly) the recommendations for a very minimal package of essential clinical services in the World Bank’s 1993 World Development Report [91]. Most countries however have shaped their packages on other bases, such as responding to national needs and expectations and assessments of available resources, though without necessarily having fully interrogated what resources are – or can be made to be – truly available, and in the absence of a clear definition and implementation of international assistance obligations. Some packages are very specific: For example, the current Chilean plan identifies 80 pathologies that are covered [92], while others only describe categories of coverage such as preventive care, outpatient and inpatient care, family planning, dental services, and drugs. Overviews of the contents are available in a variety of sources, for example the Commonwealth Fund’s profiles of 14 high-income countries [93], the Rockefeller Foundations study of a series of middle-income and low-income countries moving toward universal health coverage [94], other country studies, and country websites. While these are typically not legally binding documents they nevertheless steer national priorities with regard to health systems development and minimal services to be offered to the population.

However, the translation of these packages into effective health guarantees has been uneven, particularly within fragmented health systems with different types of health insurance and service providers where not all people receive the same benefits. Moreover there is sometimes ambiguity in how packages are defined, especially if the package identifies categories of essential benefits rather than lists of specific services. Many middle-income and lower-income countries frame essential health packages as a component of plans to move towards universal coverage, but expanding the health system to cover the population can be a slow process. In those situations the core package is more an expression of intent than a likely benefit. For example, Uganda’s minimum care packages have consistently not been met [95].

Developing the substantive content of an essential health package in line with the minimum core of the right to health therefore raises a significant dilemma. On the one hand, to hold the international community accountable for the provision of such a package in countries where assistance is needed, the description of the package must be precise enough to estimate the level of assistance needed. On the other hand, if we want the essential health package to be adapted to people’s most urgent needs – which may vary from country to country – the description of the package must be flexible enough. Such flexibility would emphasize the centrality to human rights approaches of people’s participation within national processes of adaptation that could assist in identifying health needs that must be addressed, as well as the need for systemic responses to obstacles to care such as discrimination or inaccessible services.

The need for participatory methods to define the minimum core poses challenges to an internationally defined concept of minimum core obligations that are universally applicable versus minimum core obligations that should be tailored to national settings. Certainly, in keeping with the broader human rights ambition of providing a universally applicable legal framework, General Comment 14 suggests that core obligations are intended to provide a universally applicable “bottom line” of essential health care [64]. Yet differences among countries raises questions about such a universal approach both as a substantive and practical matter. Substantively, for example, epidemiological profiles vary, and different populations within countries may have specific needs not easily determined at an international level. Practically, limited health system capacity and the time it takes to expand this capacity – training new health workers, for example – may impede even committed states from immediately being able to implement these core obligations across the entire population. Yet questions about the global versus national definition of the core are similarly applicable to the goals and targets of the post-2015 agenda. In this regard it is notable that in considering this question, the High-Level Panel has used rights as an arbiter of the necessity of setting global standards, recommending that while countries should set their own targets, goals or “global minimum standards” should be set particularly where they relate to “a universal right that every person on the planet should expect to realise by 2030, such as the eradication of extreme poverty, ending gender discrimination, education, health, food, water, energy, personal safety, and access to justice” [15].

Irrespective of how this concept is defined, the question of resources poses one of the most significant challenges to its widespread use. Certainly, the notion of minimum core obligations that place non-derogable claims on resources mounts a significant challenge to a prevailing economic common sense rooted in recession-related austerity and a longer-standing neoliberal opposition to social spending. While we acknowledge that resource constraints may impact on health care at any development level, we are nonetheless mindful that irrespective of the ‘costs’ of minimum core obligations, that scholars and policy-makers in global health should “interrogate scarcity” when it comes to essential health needs given the relatively small amounts in question relative to other domains [96]. Compare for example the 2010 peak in global health funding of USD28billion annually from all sources [97] to the USD1.9 billion daily (USD682 billion annually) spent on defence by the US alone [96,98].

We do not, however, suggest that international assistance alone can resolve the dilemma of realizing everyone’s core health entitlements. Despite the call for a minimum of 15 percent of national budgets to be allocated for health, many states including developing states are allocating 5–6 percent annually to health. States as a result are presently failing to spend the maximum of available resources on health and other economic, social, and cultural rights, such that essential health packages that states presently provide may well be needlessly constrained. Increased domestic budgetary allocations to health are crucial, as are better and more efficient use of existing health resources, including through rights-based budget analysis, adherence to principles of equity, non-discrimination and economic accessibility, participatory processes of health policy, and effective cost-effectiveness analysis. Indeed, we view cost-effectiveness analysis as a critical part of a broader human rights approach and implicit within the human rights principle of non-discrimination [64]. If states were to respect the ICESCR’s requirement that they spend “the maximum of [their] available resources” on fulfilling the rights in the Covenant, they would be investing greater resources towards fulfilling these rights, including the right to health. This, in turn, could lead to more expansive health services for all residents than states presently provide, along with the health system strengthening – including the core obligation of equitable distribution of health facilities, goods, and services – required to effectuate this guarantee. In this regard, state practices on raising and re-distributing revenue need to be closely examined, as suggested in emerging discourses around the potential revenue implications of new forms of taxation including health based taxes used only to improve health, or other revenue-raising measures such as reducing capital flight and assuring greater accountability for the use of public funds [99].

## Summary

We believe that the concept of minimum core obligations holds the potential to make an important contribution to advancing the realization of essential health needs, including within the forthcoming post-2015 health development negotiations. Yet for the minimum core concept to realize this potential requires practitioners, scholars, social groups, policy-makers and international organizations to augment the current formulation which is marked by weaknesses in key domains. We believe that a strengthened conceptualization of the minimum core concept could make a significant and innovative contribution to efforts to achieve global health equity: by providing a framework rooted in legal obligations to guide the formulation of new health development goals, providing a baseline of essential health services to be protected as a matter of right against governmental claims of scarcity and against inadequate international assistance, guiding domestic judges in assessing state compliance with right to health duties to meet basic health needs as a starting point towards the highest attainable standard of health, and empowering civil society to claim their essential health needs from domestic and global decision-makers.

## Abbreviations

ICESCR: International covenant of economic, Social and cultural rights; MDG: Millennium development goals; UN: United nations; UNCESCR: United nations committee on economic, Social and cultural rights; UNDP: United nations development program; WHO: World Health Organization.

## Competing interests

The authors declare that they have no competing interests.

## Authors’ contributions

LF completed a first draft of the manuscript, GO, AC, EL, AW, MM and EF all participated in conceptualization and assisted in drafting and revision. All authors read and approved the final manuscript.

## Pre-publication history

The pre-publication history for this paper can be accessed here:

http://www.biomedcentral.com/1472-698X/13/48/prepub
